# Ectopic Pregnancy in a Levonogestrel-Releasing Intrauterine Device User: A Case Report

**DOI:** 10.7759/cureus.18867

**Published:** 2021-10-18

**Authors:** Christina Resta, William M Dooley, Konstantinos Malligiannis Ntalianis, Sarojini Burugapalli, Munawar Hussain

**Affiliations:** 1 Obstetrics and Gynaecology, Mid & South Essex NHS Foundation Trust, Southend-On-Sea, GBR

**Keywords:** laparoscopy, female, hormonal, contraceptive agents, levonorgestrel, tubal, ectopic, pregnancy

## Abstract

Levonorgestrel-releasing intrauterine devices are considered to be a reliable contraceptive option with a low failure rate. The risk of ectopic pregnancy, however, if an unintended pregnancy occurs is significantly higher.

In this study, we present a case of a tubal ectopic pregnancy in a woman with a levonorgestrel-releasing intrauterine device in situ for one year. Our case emphasises the importance of having a high index of suspicion in women who have an intrauterine device in situ, presenting with a positive pregnancy test. We also discuss the importance of timely ultrasound examination and the management considerations of similar cases. The importance of urgent review and investigation of women with positive pregnancy test and intrauterine contraceptive device in situ, given the higher possibility of ectopic pregnancy, is highlighted by this case.

## Introduction

The use and availability of levonorgestrel-releasing intrauterine devices (LNG-IUD), including the Mirena VR and Jaydess, is increasing, being used by more than 150 million women worldwide [1]. The LNG-IUD are considered reliable contraceptive options, with a Pearl Index (number of unintended pregnancies in 100 woman-years of exposure) of approximately 0.1% [2]. Furthermore, the LNG-IUD is used in the management of menorrhagia and dysmenorrhea and is a useful option in postmenopausal women requiring oestrogen replacement, as a method to protect the endometrium from unopposed oestrogen therapy [3].

Ectopic pregnancy (EP) is described as the implantation of the embryo outside of the uterine cavity. In the general population, the overall rate of EP is 2% [4]. In cases of unintended pregnancy in women using the LNG-IUD, the chance of an EP is increased. A recent case-control study by Li et al. estimated the risk to be more than 20 times higher than women using no method of contraception [2]. Additional risk factors include in vitro fertilization, smoking, pelvic inflammatory disease (PID) and previous history of EP [5,6].

We present a case of a left tubal EP in a patient with a Mirena-IUD in situ, placed 12 months prior to the pregnancy. This case highlights the importance of considering the diagnosis of an EP in women presenting with pain or vaginal bleeding with an LNG-IUD in situ and brings up important management considerations.

## Case presentation

A healthy 36-year-old woman, who was Gravida 3, Para 2, with a history of two previous normal deliveries, attended our hospital’s Emergency Department with symptoms of abdominal pain and vaginal bleeding.

Her pain was sudden-onset, sharp left-sided pelvic pain. Her vaginal bleeding was described as light, partially soaking one pad per day, and had been ongoing for four weeks. She was a non-smoker with no history of any medical conditions or previous surgeries and no previous history of PID or EP.

A urine pregnancy test on attendance was positive. She stated that she had a Mirena-IUD inserted a year ago and that her periods had been regular and painless since then, lasting four to five days. Day one of her last menstrual period was reported to be four to five weeks prior to her attendance.

She was haemodynamically stable, with a blood pressure of 109/69 mmHg, pulse of 72 beats per minute, and temperature of 37.1°C. Physical examination revealed moderate to severe tenderness on palpation of the left iliac fossa with associated guarding and rebound tenderness. On speculum examination the strings of the IUD were visualized and the cervical os was closed with a small amount of bleeding noted.

**Figure 1:**
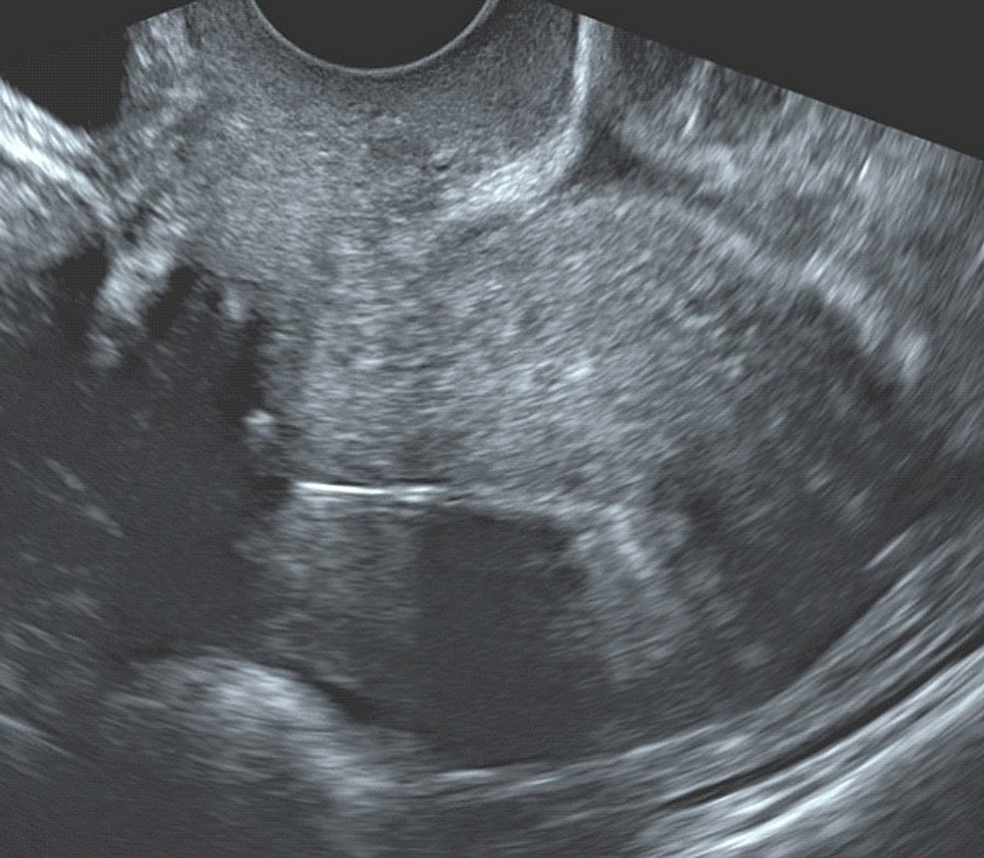
Transvaginal ultrasound showing an anteverted uterus with the Mirena-IUD correctly positioned at the fundus of the uterine cavity

**Figure 2:**
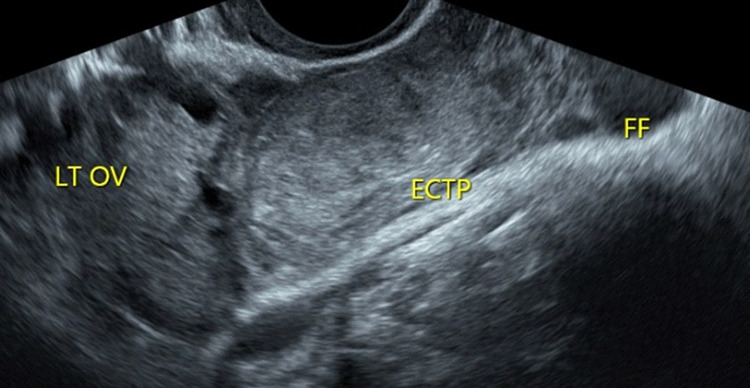
Transvaginal ultrasound scan showing a transverse view of pelvis, with annotation of the left ovary (LT OV), adjacent to the ectopic pregnancy (ECTP) and a small amount of echogenic free fluid (FF) within the adnexa.

**Figure 3:**
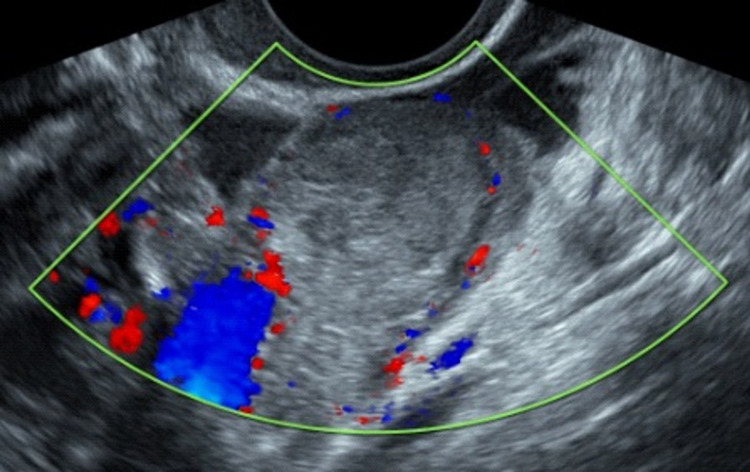
Transvaginal ultrasound scan, showing the inhomogenous mass with classical findings of a tubal ectopic pregnancy with increased surrounding vascularity.

Following these findings an urgent transvaginal ultrasound scan was performed to further evaluate the aetiology for the patient’s presentation. On transvaginal ultrasound scan, the uterus was anteverted and morphologically normal. The endometrium was regular with a Mirena IUD seen, correctly positioned in the cavity of the uterus. There was no evidence of a normally implanted intrauterine pregnancy or any retained products of conception. Both ovaries were morphologically normal. Adjacent and separate to the left ovary there was a mass that had typical appearances of a tubal EP. The Fallopian tube measured 25mm x 25mm x 21mm and the trophoblastic tissue measured 18mm x 17mm x 16mm. There was a mild amount of haemoperitoneum with some blood and no blood clots seen in the pouch of Douglas. There was no evidence of blood clots or any free fluid in uterovesical fold or pouch of Morrison. The ultrasound images are shown in Figures 1-3. 

Blood test investigations revealed a haemoglobin level of 112 g/L and serum level of beta-human chorionic gonadotropin (hCG) 150 U/L.

The options of expectant, medical and surgical management of the tubal EP were discussed. Given the patient's symptoms of significant pain, surgical management was recommended [5]. The patient consented to a laparoscopic salpingectomy. Given the IUD was seen to be correctly positioned on ultrasound scan, the option to remove it was discussed. The patient opted for removal of the Mirena-IUD as she preferred to use alternative contraception.

A diagnostic laparoscopy was performed that confirmed the above ultrasonography findings. A mass was visualised within the left Fallopian tube measuring approximately 2-3cm which had typical appearances of an EP. There was a small amount of blood in the pouch of Douglas of approximately 20mls. The Mirena-IUD was removed and a left salpingectomy performed.

The postoperative period was uneventful and a repeat pregnancy test three weeks post-surgery was reported as negative. Histopathology report confirmed the EP with no evidence of trophoblastic disease, atypia or malignancy.

## Discussion

We presented a case of a multiparous woman presenting in early pregnancy with an EP despite the presence of a Mirena-IUD. This case emphasises that women who have a positive pregnancy test with an IUD in situ should be considered high risk of EP and raises some interesting discussion points regarding the management of these women.

A large multi-national cohort study in 2015 assessed the relative contraceptive effectiveness and risk of EP in women with a LNG and copper IUD in situ [7]. It revealed that the LNG-IUD was associated with a lower risk of normally implanted and of EP compared with the copper IUD.

In our case, the Mirena-IUD was seen to be correctly positioned in the upper aspect of the uterine cavity on ultrasound examination. The risk of unplanned pregnancy in women using LNG-IUD is reported to be higher in cases where the IUD displaced either to be lower in the uterine cavity or perforated into the myometrium [7]. This emphasises the importance of assessing the location of IUD on any routine pelvic ultrasound scan and counselling women who are found to have a displaced IUD of the potential reduction in contraceptive effect.

As in our case, the commonest EP implant is within the Fallopian tube. Less common EP can be found to be implanted within the ovaries, cervix, myometrium, Caesarean section scar or abdominal cavity [8]. Studies have shown that the risk of these less-common EP is also increased in women with an IUD in place, with pathophysiology thought to be secondary to mild inflammation of uterus, nearby Fallopian tubes, and obstruction in conveyance of ovum [9,10].

In our case, the women reported that the Mirena-IUD had been in place for one year. A recent large epidemiological study showed that the risk of occurrence of an EP is higher within the first two years of insertion of IUD compared to women who have had the Mirena-IUD in for longer (5.64 per 1000 vs 2.25 per 1000, respectively) [7].

In our case, the accuracy of ultrasound was shown, in terms of the diagnosis of EP and in the quantification of haemoperitoneum. Recent studies have shown the accuracy of ultrasound diagnosis of EP to be high, with a reported positive predictive value of up to 99.4% (95% CI, 98.6-99.8%) [11]. Equally, studies have shown that semi-quantitative ultrasound assessment of the amount of haemoperitoneum on ultrasound has been shown to be positively correlated with the intraoperative findings [12]. Having accurate ultrasound findings in the location and size of the pregnancy and in the estimated volume of haemopertioneum make it an invaluable tool in guiding management decisions in cases of early pregnancy.

Furthermore in our case report, we were able to visualise the EP with a beta-hCG level of 150 U/L, thus emphasising that patients should be triaged for ultrasound, based on their symptoms and examination findings, and that serum beta-hCG levels should be used to guide management or in cases of pregnancy of unknown location [11].

In women with unintended pregnancies with an IUD in situ, the decision on whether to remove the IUD should be agreed upon with the patient after careful discussion of the risks of both routes. If the IUD is misplaced, then removing it is preferable given the ongoing suboptimal contraception provided. In cases where the IUD is correctly positioned and there is an adjacent intrauterine pregnancy seen, there has been shown to be an up to 80% chance of miscarriage if the IUD is left in situ [13]. There is therefore so there is rationale to aiming to remove the IUD as early into the pregnancy as possible; however this risk needs to be balanced with the possible increased risk of miscarriage at the time of removal. In women found to have EP, such as in our case, many women wish to have the IUD removed and to use an alternative form of contraception, given the unintended pregnancy, or may opt to keep the IUD in place. 

This case report demonstrates the need for urgent investigation for IUD users with a positive pregnancy test, given the higher risk of complications and need to clearly advise women of the different management options.

## Conclusions

Given the risk of significant morbidity in a delayed EP diagnosis, this case highlights that although unintended pregnancies are uncommon with women with IUD in place, clinicians should be aware of the higher chance of EP in women who do become pregnant. Recent advances in ultrasound have allowed for accurate and non-invasive diagnosis of EP which allows for timely information to help guide management advice.
